# Pretreatment and Acquired Drug Resistance in Children With Human Immunodeficiency Virus Type 1 in Jos, Nigeria

**DOI:** 10.1093/ofid/ofae092

**Published:** 2024-02-19

**Authors:** Augustine O Ebonyi, Jonathan Okpokwu, Holly Rawizza, Philippe Chebu, Beth Chaplin, Donald Hamel, Stephen Oguche, Oche O Agbaji, Atiene S Sagay, Phyllis J Kanki, Godwin E Imade

**Affiliations:** Department of Paediatrics, University of Jos, Jos University Teaching Hospital, Jos, Nigeria; AIDS Prevention Initiative in Nigeria–supported ISO 15189 Laboratory, Jos University Teaching Hospital, Jos, Nigeria; Department of Immunology and Infectious Diseases, Harvard T. H. Chan School of Public Health, Boston, Massachusetts, USA; APIN Public Health Initiatives, Plot 1551, Apo Resettlement, Apo District, Abuja, FCT, Nigeria; Department of Immunology and Infectious Diseases, Harvard T. H. Chan School of Public Health, Boston, Massachusetts, USA; Department of Immunology and Infectious Diseases, Harvard T. H. Chan School of Public Health, Boston, Massachusetts, USA; Department of Paediatrics, University of Jos, Jos University Teaching Hospital, Jos, Nigeria; Department of Medicine, University of Jos, Jos University Teaching Hospital, Jos, Nigeria; Department of Obstetrics and Gynaecology, University of Jos, Jos University Teaching Hospital, Jos, Nigeria; Department of Immunology and Infectious Diseases, Harvard T. H. Chan School of Public Health, Boston, Massachusetts, USA; AIDS Prevention Initiative in Nigeria–supported ISO 15189 Laboratory, Jos University Teaching Hospital, Jos, Nigeria; Department of Obstetrics and Gynaecology, University of Jos, Jos University Teaching Hospital, Jos, Nigeria

**Keywords:** children, drug resistance, HIV-1, mutations, Nigeria

## Abstract

We determined pretreatment and acquired human immunodeficiency virus (HIV) drug resistance among children with HIV type 1 (HIV-1) in Jos, Nigeria. The majority (71%) of those who failed first-line antiretroviral therapy were on a nevirapine-containing regimen. The prevalence of pretreatment (48%) and acquired (76%) HIV drug resistance mutations was high in our study. Wider access to HIV drug resistance testing after treatment failure is necessary to optimize second-line treatment options among children with HIV in Nigeria.

The burden of human immunodeficiency virus type 1 (HIV-1) drug resistance (HIVDR) is increasing among children in sub-Saharan Africa [1–3]. Drug resistance mutations (DRMs) may arise pretreatment, before the commencement of antiretroviral therapy (ART), or following exposure to antiretroviral (ARV) drugs or ART. In children, pretreatment drug resistance (PDR) occurs most commonly after exposure to ARV drugs used in the prevention of mother-to-child transmission (PMTCT) [4, 5]. The nonnucleoside reverse transcriptase inhibitors (NNRTIs) used in PMTCT are most commonly implicated in HIVDR [6]. While recent studies have shown that PDR is increasing among children in sub-Saharan Africa, additional data on how PDR impacts treatment response in programmatic settings are urgently needed. PDR has the potential to increase first-line ART failure in infants and children diagnosed with HIV, with implications for increased morbidity and mortality [5]. HIVDR mutations are well documented in other settings [7–10], but there is a paucity of data on HIVDR mutations in children in Nigeria, with one study reporting a PDR prevalence of 15.9% [3]. Monitoring of HIVDR is essential for optimizing successful treatment outcomes and safeguarding the few existing treatment options available for children [11].

This study sought to identify PDR and acquired HIVDR (ADR) in a cohort of children with HIV in Nigeria. These findings will provide preliminary data for a future larger study and may provide insight into the choice of ARVs at the time of ART initiation.

## METHODS

### Study Design

This was a retrospective paired study in which existing patient data were extracted from electronic medical record databases between February 2009 and May 2018. Clinical and laboratory data as well as archived plasma samples were used. Plasma samples of children who failed first-line ART were retrieved and paired plasma samples were tested for HIVDR, including at baseline/pretreatment (PT) and virological failure (VF) (at the point of failing ART).

### Study Location

The study was carried out at the US President’s Emergency Plan for AIDS Relief/AIDS Prevention Initiative in Nigeria–supported Paediatric HIV Clinic of the Jos University Teaching Hospital in Jos, Nigeria.

### Study Population

Children ≤16 years who were living with HIV and failed first-line, NNRTI-based ART were identified from databases.

### Operational Definitions

PDR is defined as drug resistance detected prior to initiating ART in children (some of whom may have received ARV drugs for PMTCT). ADR is defined as drug resistance detected after ART initiation at the time of VF after ART initiation [12], with VF defined as 2 consecutive viral load measurements >1000 copies/mL after at least 24 weeks on ART.

### Laboratory Methods

Samples were retrieved from storage at −80°C, HIV RNA was extracted using Qiagen viral RNA extraction kit, reverse transcribed, and amplified using Thermo Fisher HIVDR kit module 1. Purified polymerase chain reaction (PCR) products were quantified using PicoGreen dye and Fluorometer. Cycle sequencing were performed using Thermo Fisher HIVDR kit module 2. Sequences were purified using xterminator BigDye method. Sequencing was carried out with POP 7 polymer to sequence the pol gene using the ABI prism 3130 XL genetic analyzer 16 capillary model by Sanger sequencing.

### Data Analyses

Sequencing files were assembled and manually edited using Recall 2.25 software. Sequence identity matrix was performed using Bioedit software to check for contamination. HIVDR profiles were determined using the HIVdb algorithm version 8.2 and subtyping using the REGA HIV-1 subtyping tool version 3.0 on the Stanford HIVDB website [13]. Subtype classification was confirmed using clustalX and neighbor-joining phylogenetic tree in NJ-plot.

### Antiretroviral Therapy

Timing of ART initiation evolved over time and was consistent with World Health Organization (WHO) and Nigerian National ART guidelines; before 2016, specific age and immunologic criteria were utilized to determine eligibility, whereas from 2016 immediate ART initiation was recommended for all children regardless of immunologic status. Once ART was initiated, children were monitored weekly for the first month, then monthly for clinical improvement, and viral load enumeration and CD4^+^ count were checked every 6 months.

During the study period (2009–2018), based on 2010 Nigeria national guidelines [14], first-line ART among children included any one of the following regimens: zidovudine (ZDV) + lamivudine (3TC) + nevirapine (NVP), ZDV + 3TC + efavirenz (EFV), or ZDV + 3TC + abacavir (ABC); second-line regimens included ABC or tenofovir disoproxil fumarate + 3TC + lopinavir/ritonavir. Patients on dolutegravir after being rolled out in 2019 [15] were not included in this study.

## RESULTS

Among the 28 paired samples tested, 21 pairs were successfully genotyped. Of the 21 children with paired samples, 13 (62%) were female; median age at the time of enrollment into care was 6.3 (interquartile range [IQR], 1.3–10.5) years, whereas at treatment failure it was 11.6 (IQR, 7.0–13.7) years. The median CD4 cell count and viral load at VF were 515 (IQR, 245–766) cells/μL and 4.5 (IQR, 3.84–4.89) log copies/mL, respectively.

Among the successfully genotyped samples, PDR mutations were detected in 48% (10/21), while at VF 76% (16/21) had ADR.

Of the 10 children with PDR, the majority (80% [8/10]) did not have prior ARV exposure. Most of the children studied (52% [11/21]) acquired HIV infection via mother-to-child transmission (MTCT); for the remaining 10 (48%), the mode of transmission was not known as this information was not documented in their medical records/database. And of the 10 children with PDR, for 6 (60%) of them MTCT was the mode of HIV transmission while for 4 of them the mode of transmission is not known.

The majority of the children who failed first-line ART (71.4% [15/21]) were receiving NVP-containing regimens (ZDV + 3TC + NVP), while 29% (6/21) were on an EFV-containing regimen (ZDV + 3TC + EFV). The median duration on ART at VF was 2.4 (IQR, 1.5–5.0) years and the median time interval between PT and VF samples was 2.7 (IQR, 1.3–5.3) years.

### NRTI Resistance Mutations and NNRTI Resistance Mutations

The predominant nucleoside reverse transcriptase inhibitor (NRTI) and NNRTI mutations detected among the 21 children at PT and at VF are shown in Table 1.

**Table 1.** Human Immunodeficiency Virus Type 1 Drug Resistance Mutations Detected Among Children in the Various Classes of Antiretroviral Drugs

Detected Mutations	At Baseline, No., (%)	At Virological Failure, No. (%)	*P* Value
Children with detected mutations in the various classes (n = 21)	10 (48.0)	16 (76.0)	
NRTI mutations			.912
M184V	4 (19.0)	12 (57.1)	
D67N	3 (14.3)	4 (19.0)	
T215F	2 (9.5)	4 (19.0)	
K70R	2 (9.5)	3 (14.3)	
M41ML	2 (9.5)	3 (14.3)	
NNRTI mutations			.954
G190A	2 (9.5)	7 (33.3)	
Y181C	2 (9.5)	2 (9.5)	
V108VL	1 (4.8)	2 (9.5)	
Y188L	1 (4.8)	2 (9.5)	
K103N	1 (4.8)	2 (9.5)	
E138Q/A	2 (9.5)	3 (14.3)	
No. of PI mutations			
0	0 (0.0)	0 (0.0)	
No. of NRTI mutations			
0	17 (81.0)	8 (38.0)	
1	1 (4.8)	5 (24.0)	
2	0 (0.0)	2 (9.5)	
≥3	3 (14.0)	6 (28.5)	
No. of TAM mutations			
0	18 (86.0)	14 (66.7)	
1	0 (0.0)	2 (9.5)	
2	0 (0.0)	1 (4.8)	
≥3	3 (14.0)	4 (19.0)	
No. of NNRTI mutations			
0	10 (47.6)	5 (24.0)	
1	6 (28.5)	6 (28.5)	
2	1 (4.8)	6 (28.5)	
≥3	4 (19.0)	4 (19.0)	

Abbreviations: NNRTI, nonnucleoside reverse transcriptase inhibitor; NRTI, nucleoside reverse transcriptase inhibitor; PI, protease inhibitor; TAM, thymidine analogue mutation.

### HIV-1 Subtypes and Drug Resistance Mutations

**Figure 1.**
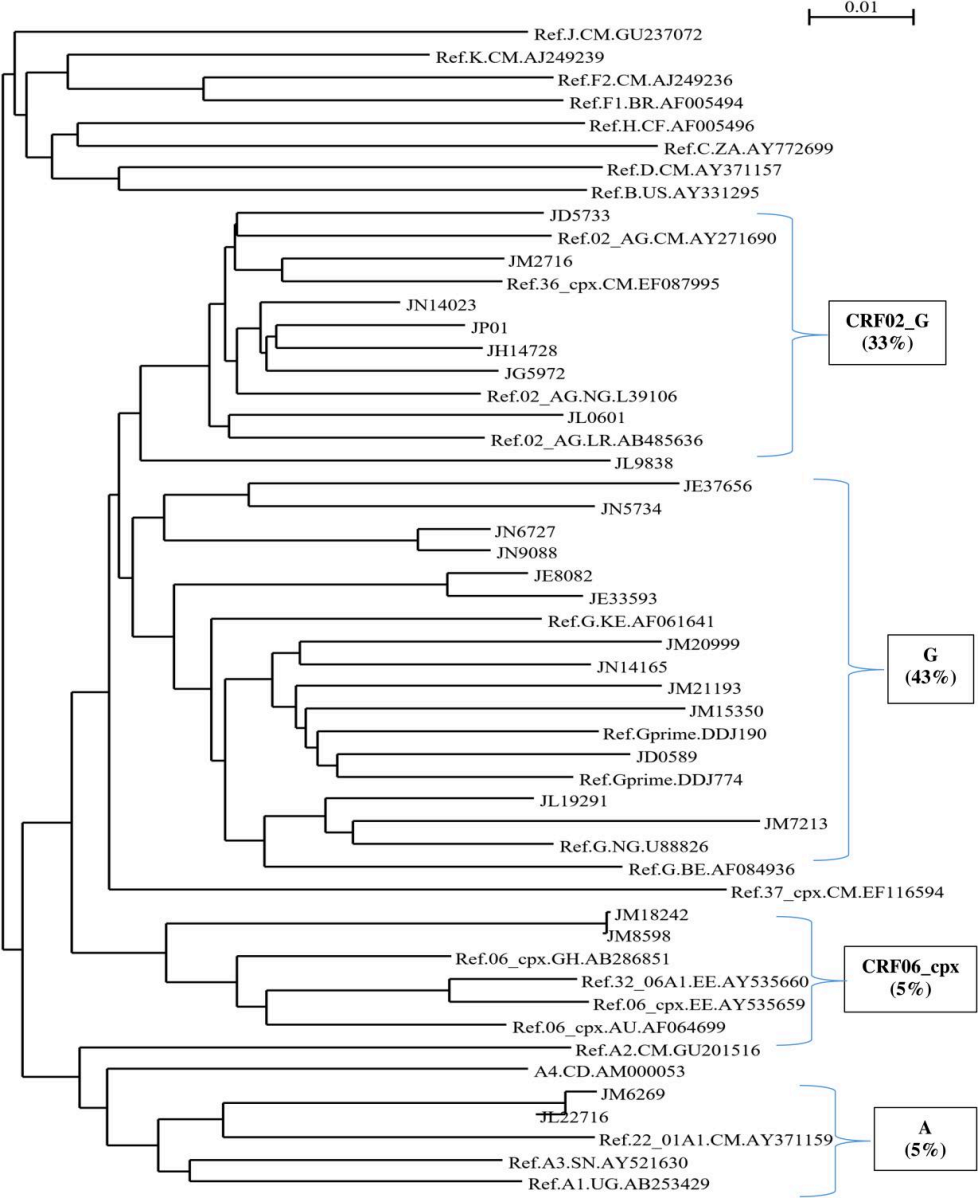
Phylogenetic tree of human immunodeficiency virus type 1 (HIV-1) subtypes in children with HIV-1 in Jos, Nigeria.

The major subtypes characterized by protease-reverse transcriptase sequencing included subtype G accounting for 43% (9 of 21), CRF02_AG for 33% (7/21), other subtypes for 24% (5/21) such as CRF06_cpx 5% (1/21) and A 5% (1/21), and recombinant forms of A and G for 14% (3/21) (Figure 1). The majority of DRMs found in this study (ie, M184V and E138A/K/Q) occurred among those with subtype G. In contrast, subtype A was more commonly associated with thymidine analogue mutations (TAMs) such as M41ML, D67N, K70R, T215FS, and G190A mutations. At VF, the TAMs detected in CRF02_AG were TAM-1 (T215F, K219E) and TAM-2 (K70R); for CRF06_cpx, the detected TAMs were D67DN and K70KR.

The total number of NRTI DRMs at PT and VF was 23 and 19, respectively, while the total number of NNRTI DRMs at PT and VF was 15 and 23, respectively.

## DISCUSSION

### The Burden of HIV Drug Resistance

The prevalence of both PDR (48%) and ADR (76%) were high in our study. The majority (80% [8/10]) of the 10 children with PDR have no known/documented prior PMTCT ARV exposure as neither they nor their mothers were enrolled in our PMTCT program. Children born to mothers who fail to disclose previous exposure to PMTCT could carry transmitted HIVDR mutations, which are known to contribute to first-line ART failure. A previous study from Lagos, Nigeria, showed a much lower prevalence of 15.9% for PDR for children without prior PMTCT ARV exposure [3]. Also, a recent systematic review of 19 studies from 13 countries in sub-Saharan African gave a pooled PDR prevalence of 12.7% among PMTCT-unexposed children [2]. The high PDR level of 48% among our PMTCT-naive children is worrying as this could further compound the problem of first-line ARV treatment failure. Such a high prevalence also calls for multicenter studies in Nigeria and for a national survey to estimate the national PDR prevalence, which may inform the modification of existing treatment guidelines, as recommended by the WHO when PDR levels exceed 15% [16].

In a systematic review involving 26 countries and 36 studies of 1980 children with both NRTI and NNRTI mutations, a wide range of ADR prevalence was observed including 0% in France, 2% in the United States, 7% in South Africa, 30% in Malawi, 67% in Mali, and 100% in Uganda [1]. In our study, ADR was 76%, with mutations that confer resistance to both NRTI and NNRTI medications, but none for protease inhibitors. ADR is generally higher in resource-limited settings compared to resource-rich countries, which have well-established drug resistance testing and monitoring systems.

### NRTI Resistance Mutations

In our study, NRTI resistance mutations were present at both PT and VF, with the most prevalent being M184V (57.1%) and D67N (19.0%) at VF. M184V increased by 38.1% from PT to VF, while other mutations such as D67N, T215F, K70R, and M41ML persisted from PT to VF. A recent multicenter prospective cohort study of African children with VF reported 69.7% had M184VI, higher than in our study [17]. Accumulation of DRMs can be prevented/reduced with HIVDR testing to detect mutations early and guide choice of first-line regimens. The lack of testing could have contributed to high levels of M184V mutations at VF in our study (Table 1). ZDV was part of the preferred first-line ART regimen [14] during this study and may select for TAMs such as M41L, D67N, L210W, T215Y/F, and K219Q/E, which are known to impact susceptibility of most NRTIs excluding lamivudine and emtricitabine [18]. In our setting, children failing first-line ART are blindly switched to second-line ART without HIVDR testing, which could further compound the problem of HIVDR.

### NNRTI Resistance Mutations

The predominant NNRTI DRMs were G190A (9.5% at PT and 33.3% at VF) and Y181C (9.5% at PT and at VF) with a 3.5-fold increase in G190A at VF. The majority (71.4%) of children who failed first-line ART were those on NVP-containing regimens and the majority (80%) did not have recorded/documented prior PMTCT ARV exposure. A previous study in Nigerian adults reported that the most common NNRTI mutations were Y181C (49.7%), K103N (36.4%), G190A (26.3%), and A98G (19.5%) [19]. Our findings are consistent with studies reporting that the NNRTI mutations (G190A, K103N, and Y181C) are most frequently associated with HIVDR in long-term NVP-containing ART [20] and may result in failure of other NNRTI-containing regimens [21], and that NVP resistance mutations resulted after repeated use of NVP for the prevention of perinatal HIV transmission [22].

### Study Limitations

Our study sample size of 21 children was small and not representative of the clinic cohort of children with HIV-1 on ART, so the 47.5% PDR prevalence may likewise not be representative. Also, mothers having had PMTCT or other ART exposure before enrolling at our center could have contributed to the high PDR prevalence.

## CONCLUSIONS

The prevalence of both PDR and ADR was high in our setting and could compromise future second-line ART regimens. There is an urgent need for routine HIVDR testing and monitoring among children with HIV-1 in Nigeria.

## Notes

***Author contributions.*** A. O. E., G. E. I., H. R., and J. O. contributed to study design, statistical analysis, and drafting of the manuscript. A. O. E., J. O., S. O., G. E. I., H. R., A. S. S., O. O. A., B. C., D. H., P. C., and P. J. K. contributed to data acquisition and interpretation, as well as critical revision for intellectual content. All authors reviewed and approved the final version of the manuscript.

***Acknowledgments.*** We thank all the children who participated in this study; AIDS Prevention Initiative in Nigeria (APIN) and Jos University Teaching Hospital (JUTH) for permission to use the patients’ data; Bola Olatunde for data abstraction; and Titus Obadiah and Mangai Yakubu for sample handling and transportation.

***Patient consent.*** Written informed consent for secondary use of data for research was obtained from the parents/guardians of the children at the time of enrollment into HIV care and treatment, and the study was approved by the Ethics Committee of JUTH.

***Financial support*.** This work was supported by a STAMINA Grant (Support of Training and Mentoring in Nigeria for Academics) through funding from the Fogarty International Center Office of the Director, the National Institute of Neurological Disorders and Stroke, and the National Institute of Nursing Research of the National Institutes of Health (grant number D43 TW010130). The US Department of Health and Human Services, Health Resources and Services Administration (U51HA02522- 01-01) and the Centers for Disease Control and Prevention (PS 001058) supported HIV/AIDS treatment and care services at APIN/JUTH.
